# Regulation of vitamin D metabolizing enzymes in murine renal and extrarenal tissues by dietary phosphate, FGF23, and 1,25(OH)_2_D_3_

**DOI:** 10.1371/journal.pone.0195427

**Published:** 2018-05-17

**Authors:** Larissa Kägi, Carla Bettoni, Eva M. Pastor-Arroyo, Udo Schnitzbauer, Nati Hernando, Carsten A. Wagner

**Affiliations:** 1 Institute of Physiology, University of Zurich, Zurich, Switzerland; 2 National Center for Competence in Research NCCR Kidney.CH; Charles P. Darby Children’s Research Institute, UNITED STATES

## Abstract

**Background:**

The 1,25-dihydroxyvitamin D_3_ (1,25(OH)_2_D_3_) together with parathyroid hormone (PTH) and fibroblast growth factor 23 (FGF23) regulates calcium (Ca^2+^) and phosphate (Pi) homeostasis, 1,25(OH)_2_D_3_ synthesis is mediated by hydroxylases of the cytochrome P450 (Cyp) family. Vitamin D is first modified in the liver by the 25-hydroxylases CYP2R1 and CYP27A1 and further activated in the kidney by the 1α-hydroxylase CYP27B1, while the renal 24-hydroxylase CYP24A1 catalyzes the first step of its inactivation. While the kidney is the main organ responsible for circulating levels of active 1,25(OH)_2_D_3_, other organs also express some of these enzymes. Their regulation, however, has been studied less.

**Methods and results:**

Here we investigated the effect of several Pi-regulating factors including dietary Pi, PTH and FGF23 on the expression of the vitamin D hydroxylases and the vitamin D receptor VDR in renal and extrarenal tissues of mice. We found that with the exception of Cyp24a1, all the other analyzed mRNAs show a wide tissue distribution. High dietary Pi mainly upregulated the hepatic expression of Cyp27a1 and Cyp2r1 without changing plasma 1,25(OH)_2_D_3_. FGF23 failed to regulate the expression of any of the studied hydroxylases at the used dosage and treatment length. As expected, renal mRNA expression of Cyp27b1 was reduced and Cyp24a1 was increased in response to 1,25(OH)_2_D_3_ treatment. However, the 25-hydroxylases were rather unaffected by 1,25(OH)_2_D_3_ treatment.

**Conclusions:**

The analyzed vitamin D hydroxylases are regulated in a tissue and treatment-specific manner.

## Introduction

The hormone 1,25-dihydroxyvitamin D_3_ (1,25(OH)_2_D_3_), together with fibroblast growth factor 23 (FGF23), parathyroid hormone (PTH) and dietary phosphate (Pi), plays a key role in homeostasis of Pi. (for review see [1–3]) Additionally, these three hormones regulate each other levels via positive and negative feedback loops, which makes difficult to discriminate individual effects. In particular, 1,25(OH)_2_ vitamin D_3_ levels are blunted in response to high dietary Pi [4], high FGF23 [5] and high 1,25(OH)_2_ vitamin D_3_ itself [6]. On the other hand, while high dietary Pi and high FGF23 promote renal excretion of Pi, high 1,25(OH)_2_ vitamin D_3_ promotes intestinal absorption of Pi. 1,25(OH)_2_D_3_ is also a regulator of calcium (Ca^2+^) homeostasis and in addition it may play an important role in degenerative diseases, cardiovascular disease, and cancer [7]. The vitamin D_3_ precursor 7-dehydrocholesterol is converted to previtamin D3 by the exposure of the epidermis and dermis to ultraviolet B radiation (290–320 nm). In a temperature-depending reaction, this previtamin is isomerized to vitamin D_3_ before being transported to the blood circulation where it binds to the vitamin D-binding protein (DBP) [8]. Vitamin D_2_ and D_3_ as well as their metabolites 25-hydroxyvitamin D_2_, and D_3_ are also absorbed from the diet in the intestine [9]. The fat-soluble compounds are absorbed in micelles by enterocytes and transported by chylomicrons into the lymphatic system and further into the circulation [10].

To become the active form, vitamin D_2_ and D_3_ must undergo two modification steps catalyzed by hydroxylases of the cytochrome P450 (Cyp) family. These enzymes possess specific domains for the binding of a heme group and for the interaction with electron-transferring proteins [11]. Both vitamin D_2_ and D_3_ are metabolized in a similar way; for simplification, only the vitamin D_3_ modification reactions are summarized next. The first modification step takes place in the liver where vitamin D_3_ is converted to 25-hydroxyvitamin D_3_ (25(OH)D_3_) by hydroxylation at C-25. The hepatic 25-hydroxylases are the mitochondrial CYP27A1 and the microsomal CYP2R1 [12]. CYP27A1 is expressed not only in the liver but is widely distributed and it hydroxylates vitamin D_3_ but not D_2_ [13]. Most probably CYP27A1 is not the major 25-hydroxylase since *Cyp27a1* knockout mice have a two- to threefold increase in serum 25(OH)D_3_ level, suggesting a compensatory role of CYP2R1 [14, 15]. Furthermore, deletion of the *Cyp27a1* gene does not result in rachitic phenotypes in mice [16]. On the other hand, the microsomal CYP2R1 is mostly expressed in liver and testis and it is considered to play a major role in 25-hydroxylation of vitamin D_2_ and D_3_, since mutations or depletion of Cyp2r1 lead to reduced 25(OH)D_3_ levels [15, 17]. Nevertheless, additional enzymes with 25-hydroxylase activities probably exist as *Cyp2r1*^*-/-*^*/Cyp27a1*^*-/-*^ double knockout mice show similar 25(OH)D_3_ levels in plasma as *Cyp2r1* knockout animals [15]. The second step of modification takes place mainly in the proximal tubule of the kidney and it is mediated by the mitochondrial 1α-hydroxylase encoded by the CYP27B1 gene, resulting in the production of the fully active 1,25(OH)_2_D_3_ [13, 18]. In contrast to the 25-hydroxylation, the renal CYP27B1 is most probably the only enzyme with 25(OH)D_3_ 1α-hydroxylation activity since inactivating mutations in the CYP27B1 gene abolish the production of 1,25(OH)_2_D_3_ and are the cause for vitamin D-dependent rickets type I (also known as pseudovitamin D-deficiency rickets) [19]. Both 25(OH)D_3_ and 1,25(OH)_2_D_3_ are degraded by the mitochondrial CYP24A1, that catalyzes their 24- or 23-hydroxylation, finally resulting in either calcitroic acid or 1α,25-dihydroxyvitamin D-26,23-lactone, respectively [12, 20]. *Cyp24a1* knockout mice highlighted the important role of this hydroxylase in the catabolism of vitamin D, as these mice showed severe hypercalcemia with about 50% perinatal mortality [21]. Also in humans loss-of-function mutations in the *CYP24A1* are a main cause of the impaired vitamin D catabolism and lead to idiopathic infantile hypercalcemia or hypercalciuric nephrocalcinosis and nephrolithiasis [22, 23].

Both renal CYP27B1 and CYP24A1 are highly regulated. Thus, PTH stimulates the expression of CYP27B1 in the kidney, whereas FGF23, high Ca^2+^ or Pi levels and 1,25(OH)_2_D_3_ itself downregulate it. In contrast, 1,25(OH)_2_D_3_ as well as FGF23 strongly induce the expression of CYP24A1, whereas PTH reduces its expression by stimulating the degradation of its mRNA [13]. Moreover, while PTH enhances the production of 1,25(OH)_2_D_3_, 1,25(OH)_2_D_3_ in turn inhibits the production of PTH either directly at the transcription level of the *PTH* gene or indirectly by increasing Ca^2+^ absorption in the intestine and stimulating the expression of Ca^2+^ sensing receptors [24, 25]. Similarly, FGF23 suppresses the production of 1,25(OH)_2_D_3_, but 1,25(OH)_2_D_3_stimulates the synthesis of FGF23 in bones [26].

In addition to the classical activation pathway described above (CYP27A1/CYP2R1 and CYP27B1-mediated hydroxylations), vitamin D_3_ and D_2_ can be hydroxylated by the mitochondrial CYP11A1/P450scc [27]. The first and major product of this last reaction is 20-hydroxyvitamin D_3_ (20(OH)D_3_), which can be further metabolized by CYP271 and CYP24A1. Many of the products of this additional pathway where detected in human serum and where shown to be biologically active; for review see [28].

The active 1,25(OH)_2_D_3_ exerts most of its biological functions via the binding to the vitamin D receptor (VDR). The ligated receptor recognizes and binds to the vitamin D response elements (VDRE) in target genes. This enables the formation of a heterodimer of the VDR and the retinoid X receptor (RXR). The formed complex recruits multiple coregulators that possess the ability to induce changes in gene expression of vitamin D target genes; for review see [3].

While renal expression and regulation of the vitamin D related hydroxylases are well documented, their extrarenal expression and local regulation are still poorly understood. In this regard, the catabolic CYP24A1is found in vitamin D target tissues including bone and intestine, while the activating 1α-hydroxylase CYP27B1 has been reported in skin, prostate, parathyroid gland, pancreatic islets, testes, placenta and cells of the immune system like macrophages and dendritic cells [13, 20]. This distribution of the 1α-hydroxylase in extrarenal tissue, as well as the association of vitamin D with human granuloma-forming diseases such as sarcoidosis or tuberculosis bring forward the argument of locally produced 1,25(OH)_2_D as a paracrine/autocrine system [29, 30].

Here, were analyzed the pattern of expression of the above mentioned vitamin D related hydroxylases (CYP27A1, CYP2R1, CYP27B1 and CYP24A1) as well as the VDR in murine renal and extrarenal sites, and their regulation by factors that alter Pi metabolism, in order to study whether or not regulation of 1,25(OH)_2_ vitamin D_3_ is achieved by similar mechanisms regardless of the original signal.

## Methods

### Animal experiments

The study was approved by the local Veterinary Authority (Kantonales Veterinäramt Zürich Approval number: 05/2013). Male NMRI and C57BL/6 mice (8–10 weeks) were purchased from Janvier Labs (France) and kept on standard diet (0.80% phosphate, 1.05% calcium, 1000 IU/kg vitamin D_3_, Promivi Kliba AG, CH) until the start of the experiments. Dietary phosphate: two randomized groups (each n = 6) of 10-week-old NMRI male mice were fed ad libitum for seven days with either high (1.2%, HPD) or low (0.10%, LPD) phosphate diet (Promivi Kliba AG, CH) with free access to water. On day eight all twelve animals were sacrificed and samples were collected as described below. Values obtained with LPD-fed mice were considered as reference. FGF23 and vitamin D_3_ treatments: two randomized groups (each n = 6) of 12 week-old NMRI male mice were injected intraperitoneally either with FGF23 (R&D Systems, Bio-techne AG, UK) (40 μg/kg body weight diluted in 9% PBS/0.1% BSA, 125mM NaCl and 10% ethanol) or with vehicle alone as control. The mice were injected intraperitoneally once a day for two consecutive days and were then sacrificed on the third day; samples were collected as described below. 1α,25(OH)_2_D_3_ (Sigma-Aldrich, Buchs, CH) was first dissolved in 100% ethanol to make 1 mM stocks (0.41664 μg/μl). Four randomized groups (each n = 6) of 10-week-old C57BL/6 male mice were injected intraperitoneally either with 1,25(OH)_2_D_3_ (4 μg/kg body weight diluted in 1.5% ethanol and 98.5% peanut oil) or with vehicle alone as control. The mice were injected either once and sacrificed 14 hours later (short-term treatment) or on day 0 and 2 and sacrificed on day 4 (long-term treatment).

For sample harvesting, each mouse was anaesthetized in an isoflurane chamber and kept anaesthetized using a nose mask with flowing isoflurane/O_2_. The abdomen was opened and the urinary bladder was emptied to collect spot urine. Urine was centrifuged at 10’000 rpm for 10 minutes and the supernatant was stored at -20 °C. A small piece of pancreas was cut and snap-frozen in liquid nitrogen and stored at -80 °C. Blood was collected by cardiac puncture of the left ventricle with heparin-prerinsed 1-ml syringes and 22G x 1 ¼ inch needles (B Braun Medical AG, CH). Plasma was obtained by centrifuging the blood sample for 7 minutes at 7’000 rpm at 4 °C and aliquots were stored at -80 °C. Upon extraction of blood, each animal was then perfused with 20 ml PBS directly by cardiac puncture of the left ventricle. The following organs and tissues were then collected, frozen in liquid nitrogen, and stored at -80 °C for further analysis: decapsulated kidney, liver, abdominal fat, brain, femur, small intestine and colon. The intestinal tissue was washed with cold 0.9% sodium chloride, everted and scraped. The mucosa was frozen in liquid nitrogen and stored at -80 °C for further analysis.

### Phosphate, calcium and creatinine measurements

Phosphate in spot urine and plasma was measured by the Fiske and Subbarow method. Calcium in spot urine and plasma was quantified using the QuantiChrom^™^ Calcium Assay Kit (BioAssay Systems, USA), following the manufacturers’ protocol. Urinary creatinine determination was performed following Jaffe’s method.

### PTH, FGF23 and 1,25(OH)_2_D_3_ measurements

PTH concentrations as well as human and mouse intact FGF23 levels in plasma were measured by ELISA (Immunotopics Inc., USA). Quantitative determination of 1,25(OH)_2_D_3_ in plasma was performed by radioimmunoassay (Immunodiagnostic Systems, UK). This kit partially cross-reacts with 1,25(OH)_2_D_2_. In all cases, measurements were done following the manufacturers’ protocol.

### Semi-quantitative real time RT-PCR

Kidney and intestinal mucosa were homogenized in RLT buffer (Qiagen Science, DE) supplemented with 1% β-mercaptoethanol. Bone marrow from femur samples was removed by flushing bones with 0.9% sodium chloride. Bones were then smashed in a mortar before being homogenized in QIAzol lysis buffer (Qiagen Science DE). Liver, abdominal fat tissue and brain samples were also homogenized in QIAzol lysis buffer. RNA from homogenates was extracted using the RNeasy Mini Kit (Qiagen Science, DE). cDNA was obtained by reverse transcription of the isolated RNA using TaqMan^®^ Reverse Transcription kit (Thermo Fisher Scientific, CH). RNA expression of several genes of interest was quantified by semi-quantitative real time PCR (RT-qPCR) using gene-specific primers (25 μM) and probes (5 μM) (S1 Table) and the KAPA PROBE FAST qPCR Kit Master Mix (2x) Universal (Kapa Biosystems, USA). Amplification of cDNA was performed in a 7500 Fast System cycler (Applied Biosystems CH). Glyceraldehyde 3-phosphate dehydrogenase (Gapdh) was used as housekeeping gene to normalize RNA expression of the target genes in all tissues except in bone for which hypoxanthine phosphoribosyl-transferase (Hprt) was used, since the expression of Gapdh was altered by some of the treatments. At a given threshold (Ct) the cycle number was measured and normalized by the housekeeping gene as following: $2^{{(Ct}_{\left(control\right)}-{Ct}_{\left(gene\right)})}$, resulting in the relative gene expression.

### Total renal protein extraction

Total protein extraction was achieved by homogenizing half a kidney in 300 μl RiPa buffer, cotaining50 mM TrisHCl pH 7.4, 150 mM NaCl, 1% NP-40, 0.5% Na-Deoxycholate, 10 μl/ml cOmplete^™^ EDTA-free Protease inhibitor cocktail (Roche Diagnostics AG, CH) and 2 mM phenylmethylsulfonyl fluoride. Tissue samples were homogenized using MagNa Lyser Green Beads and a Precellys^®^ 24 Homogenizer. The samples were then centrifuged for 10 minutes at top speed at 4 °C and the supernatants were collected. Protein concentration was determined with a BioRad DC Protein Assay (BioRad Laboratories Inc., USA).

### Western blot

Aliquots of homogenates containing 20 μg of total protein were mixed with 4x Laemmli sample buffer supplemented with 400 mM dithiothreitol (Thermo Scientific, CH), incubated for 2 minutes at 90 °C, separated into a 10% acrylamide SDS-PAGE and further transferred to a nitrocellulose membrane (Immobilon^®^-P Transfer Membrane, Merck Millipore CH) by wet transfer. The membranes were blocked for 30 minutes at room temperature (RT) with Tris buffered saline supplemented with 0.1% Tween 20 (TBST) and 5% fat-free milk and further incubated overnight at 4 °C with available primary antibodies against NaPi-IIa (dilution 1:3’000, University of Zurich, CH [31]), Klotho (dilution 1:1’000, TransGenic Inc., JP), Cyp24a1 (dilution 1:1’000, Proteintech ^™^, UK), Vdr (dilution 1:500, Santa Cruz Biotechnology Inc., USA) and β-actin (dilution 1:12’000, Sigma Aldrich, CH). After 3 washing steps with TBST, the membranes were again blocked and incubated for 2 hours at RT with the appropriate secondary (anti-mouse, -rabbit, -rat) antibody linked to horseradish peroxidase (HRP) (GE Healthcare, UK; R&D Systems, USA). Upon 3 washing steps with TBST, membranes were incubated with a HRP substrate (Merck Millipore CH) for 5 minutes. The chemiluminescent signal was detected using the Luminescent Image Analyzer LAS-4000. Quantitiy One^®^ (Version 4.6.1, BioRad) was used for the densitometric quantification and the expression of the protein of interest was normalized to the expression of β-actin.

### Statistical analysis

Data are presented as mean ± standard deviation and statistical analysis were performed with GraphPad Prism Version 5.02, comparing two groups by unpaired Student’s t-Test. P values ≤0.05 were considered as statistically significant.

## Results & discussion

### High dietary Pi load increases the expression of 25-hydroxylases in the liver

**Fig 1:**
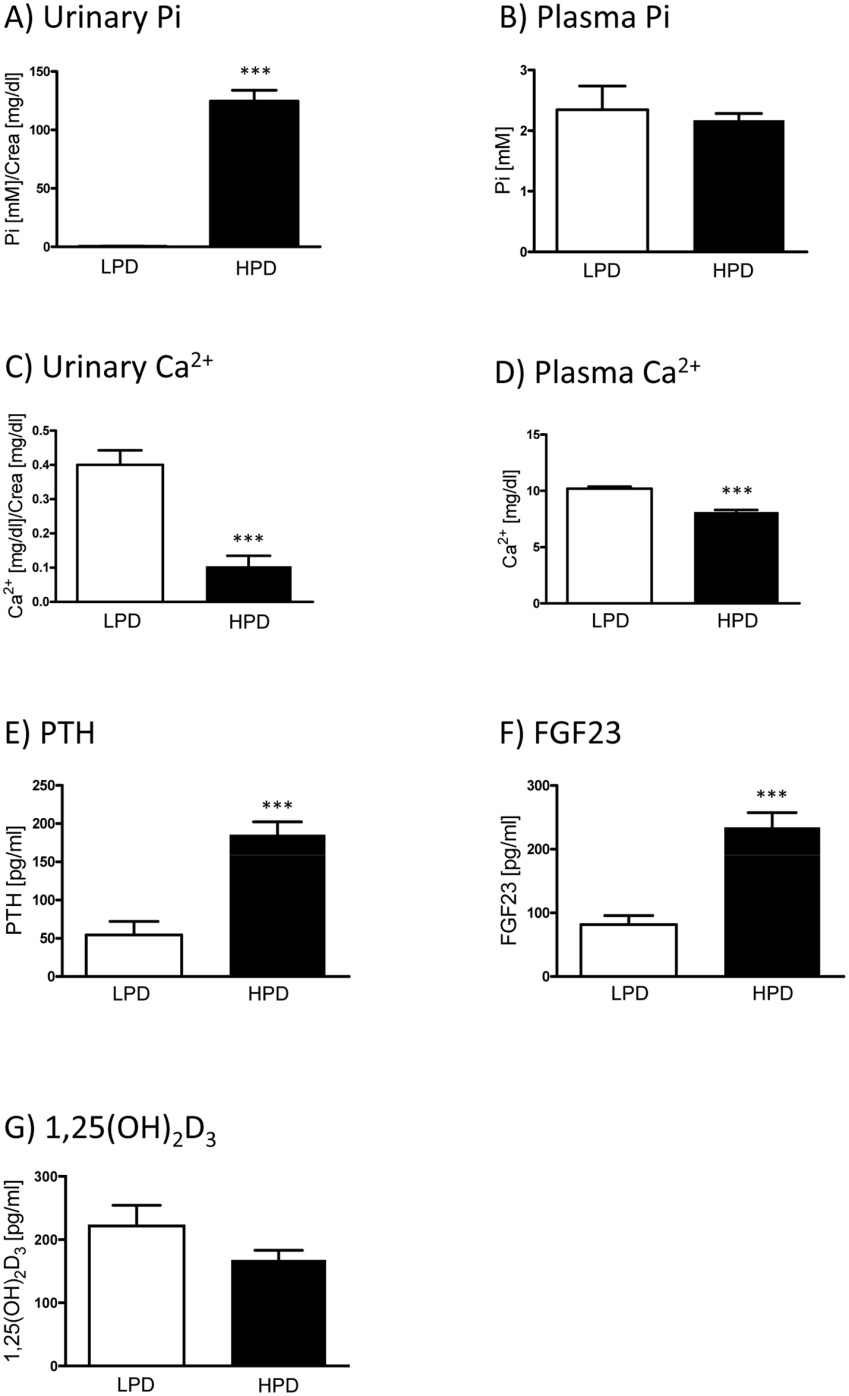
Effect of dietary Pi intake on mineral and hormonal levels. Urinary and plasma concentrations of Pi **(A and B)** and Ca^2+^ **(C and D)** as well as plasma levels of PTH **(E)**, intact FGF23 **(F)** and 1,25(OH)_2_D_3_ **(G)** in samples from mice fed either high (1, 2%, HPD) or low (0.1%, LPD) Pi diet for seven days. Graphs show mean ± SD (n = 6/group). Differences between both dietary groups were analyzed by unpaired student’s t-test with significant p values indicated as: *** p < 0.0001.

Mice were kept for 5 days on either low (0.1%) or high (1.2%) Pi diets. To test whether the dietary protocols had produced the expected systemic adaptations, the concentrations of Pi and Ca^2+^ in urine and plasma as well as the levels in plasma of several hormones were quantified first. Mice fed high Pi diet (HPD) had significantly higher excretion of urinary Pi compared to mice fed low Pi diet (LPD) (Fig 1A), while plasma Pi levels were similar in both groups (Fig 1B). In addition, HPD led to significantly lower urinary and serum Ca^2+^ levels (Fig 1C and 1D). These were the expected effects, as the kidney responds to high Pi intake with increased urinary Pi excretion to maintain normal serum phosphate [32], and it has been reported that a diet high in Pi decreases urinary and serum Ca^2+^ levels [33]. Mice adapted to a HPD had significantly higher concentrations of intact PTH as well as intact FGF23 in plasma, compared to mice fed LPD (Fig 1E and 1F), whereas plasma 1,25(OH)_2_D_3_ levels did not differ significantly between the two groups (Fig 1G). FGF23 and PTH regulate Pi homeostasis by reducing the capacity of the kidney to reabsorb Pi, thus inducing phosphaturia; reciprocally high plasma Pi increases the levels of FGF23 and PTH [34, 35]. The low urinary excretion of Ca^2+^ observed in the HPD group can further be explained by the binding of intestinal Ca^2+^ by intestinal Pi reducing its absorption and by the high PTH levels, as PTH enhances the reabsorption of Ca^2+^ in the kidney [36]. Collectively, these data indicate that the dietary challenge had produced the expected mineral and hormonal adaptations. The synthesis of 1,25(OH)_2_D_3_ is regulated by PTH and FGF23, with PTH stimulating the synthesis and inhibiting the degradation of 1,25(OH)_2_D_3_, whereas FGF23 downregulates the production and induces the catabolism of 1,25(OH)_2_D_3_ in the kidney [24]. Therefore, the fact that similar levels of 1,25(OH)_2_D_3_ were detected in the HPD and LPD groups may be explained based on the high PTH and FGF23 levels in the HPD group, as both hormones might counterbalance their effects on the synthesis and catabolism of 1,25(OH)_2_D_3_.

**Fig 2:**
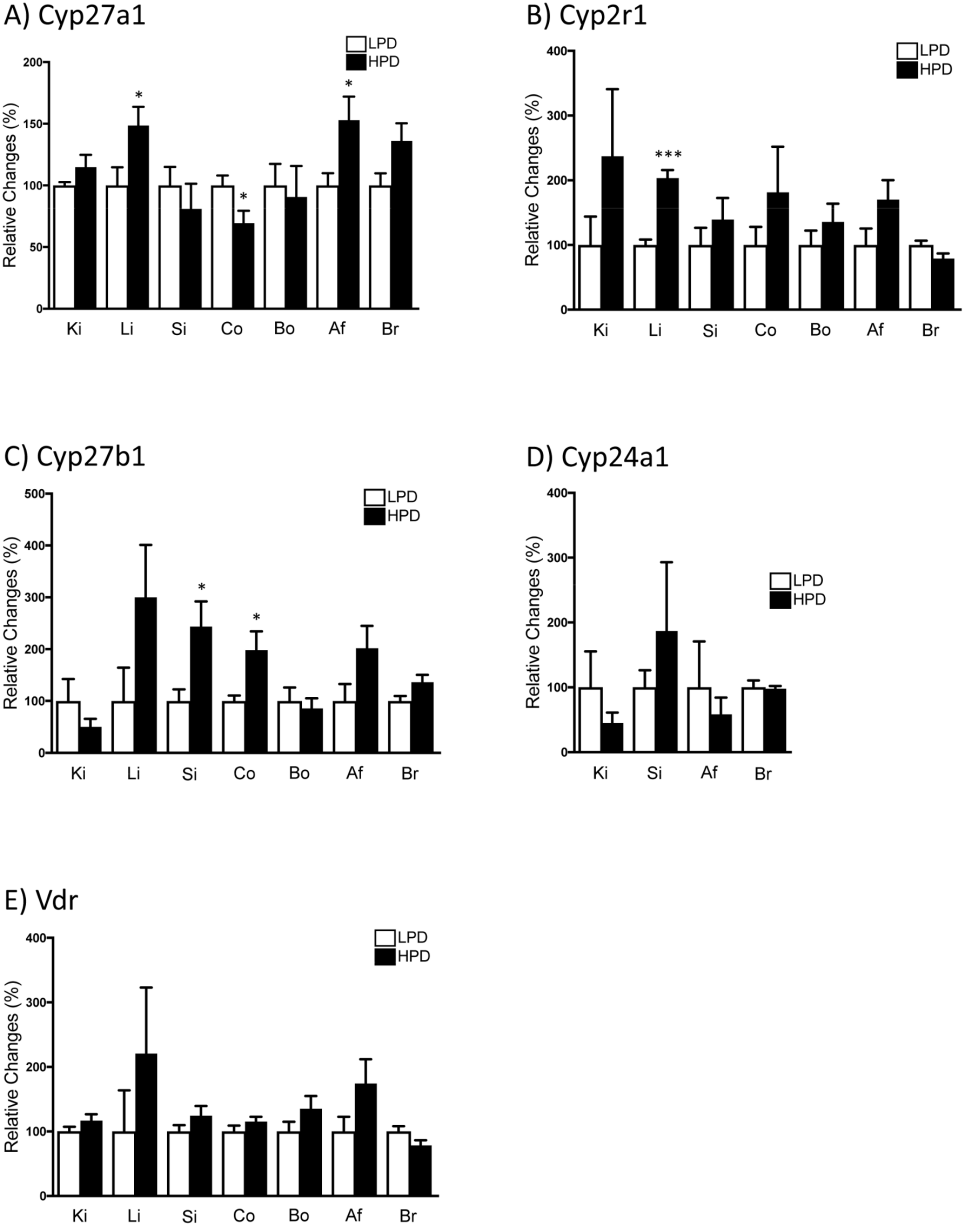
Dietary Pi intake changed extrarenal expression of 25- & 1α-hydroxylases. mRNA expression of *Cyp27a1* **(A)**, *Cyp2r1* **(B)**, *Cyp27b1* **(C)**, *Cyp24a1* **(D)** and *Vdr* **(E)** was measured in kidney (Ki), liver (Li), small intestine (Si), colon (Co), bone (Bo), abdominal fat (Af) and brain (Br) samples of mice fed either high (HPD; 1.2% Pi) or low (LPD; 0.1% Pi) Pi diet for seven days. Expression of the mRNA of interest was normalized to *Gapdh* (*Hprt* was used for normalization in bone). Graphs represent mean ± SD (n = 6/group) of relative changes. Differences between both dietary groups were analyzed by unpaired student’s t-test with significant p values indicated as: * p ≤ 0.05 and *** p < 0.0001.

The 25-hydroxylase *Cyp27a1* mRNA was detected in all analyzed organs (S2 Table), though it was especially abundant in liver, colon, bone, and abdominal fat. Its expression was higher in liver and abdominal fat of mice fed HPD compared with LPD, while the opposite pattern of expression was detected in colon (Fig 2A). The other main 25-hydroxylase, namely *Cyp2r1*, was also detected in all tested tissues (S2 Table), with liver and bone showing the highest levels. Its hepatic abundance was also higher in mice fed HPD compared to mice fed LPD (Fig 2B). This suggests that high dietary Pi intake may regulate the expression of the hepatic and fat 25-hydroxylases. Further studies are required to clarify whether this is a direct effect or whether it requires PTH or FGF23, since both hormones were elevated. As low Ca^2+^ levels were measured in plasma of mice fed HPD, Ca^2+^ could also lead to local stimulation of 25(OH)D_3_ production in liver and adipose tissue. The fact that the expression pattern in colon is the opposite to liver and fat indicates that the regulation of vitamin D metabolizing enzymes might be cell and tissue specific. Although 1α-hydroxylase *Cyp27b1* mRNA was detected in all analyzed organs, its expression was especially abundant in kidney and bone whereas it was very low in liver, small intestine and abdominal fat (S2 Table). *Cyp27b1* abundance was higher in the small intestine and colon of mice fed with HPD compared to those fed a LPD (Fig 2C). It has been reported that in the absence of potent stimuli, the expression of the 24-hydroxylase is low, even below the detectable level, in vitamin D target tissues [37]. Accordingly, and with the only exception of kidney, the gene expression of Cyp24a1 was barely detectable (S2 Table). Furthermore, dietary Pi seemed not to affect its expression in any of the analyzed organs (Fig 2D). Neither renal *Cyp27b1* nor renal *Cyp24a1* were regulated by dietary Pi, although dietary effects were shown in C57BL/6 mice [38, 39]. Whether this lack of regulation is a strain-specific feature of NMRI mice needs further investigation. *Vdr* mRNA was detected in all organs of interest except liver, where the expression was too low for consistent quantification, with the higher levels detected in bone, colon small intestine and kidney (S2 Table). *Vdr* expression was not regulated by dietary Pi in any of the analyzed tissues (Fig 2E). It is known that 1,25(OH)_2_D_3_ enhances the expression of its own receptor [40]; thus the similar *Vdr* expression detected in both dietary groups may reflect their similar 1,25(OH)_2_D_3_ plasma levels.

**Fig 3:**
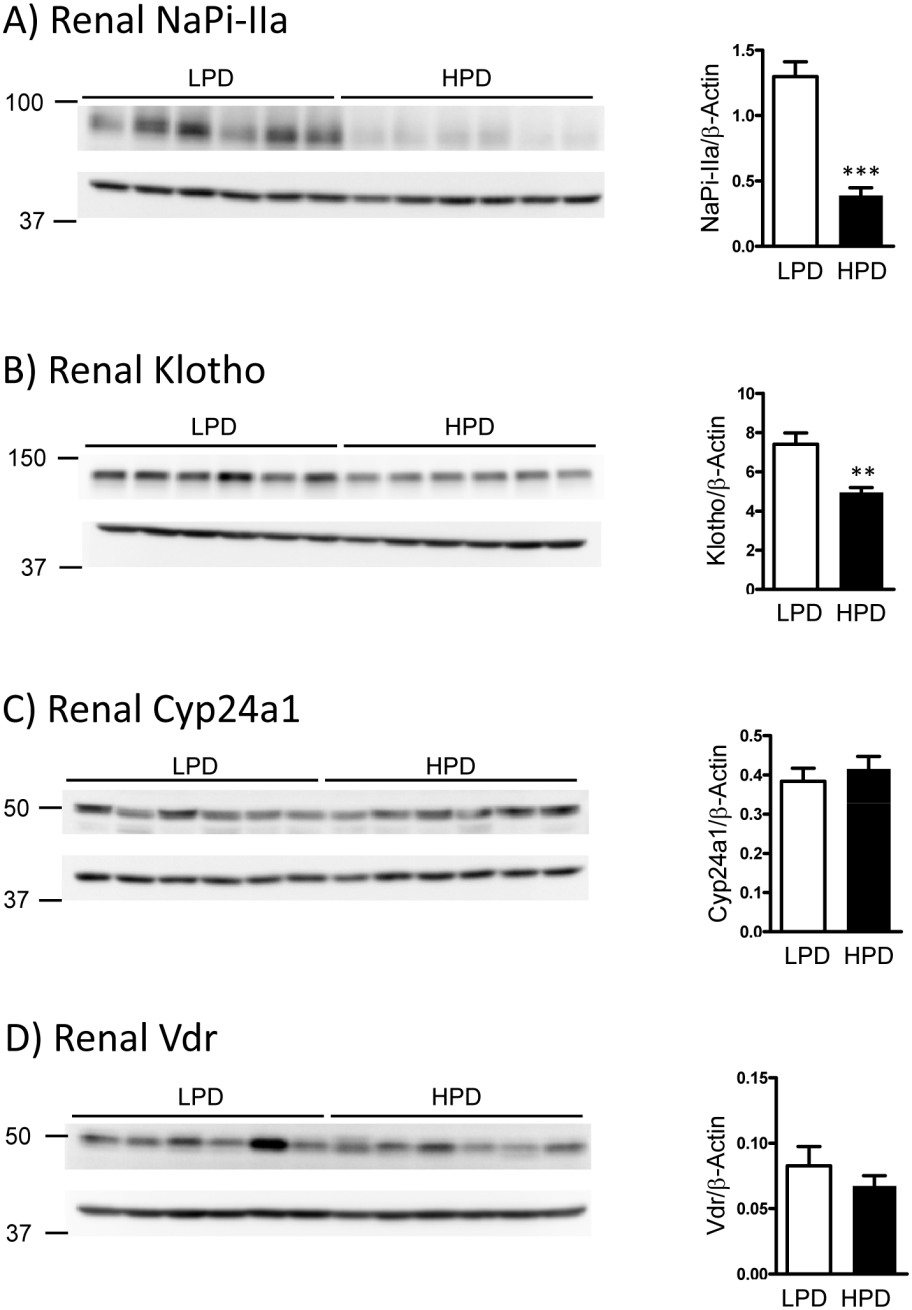
Selective adaptation of renal protein expression to dietary Pi intake. Representative western blots of total renal protein homogenates from mice fed high (HPD) or low (LPD) Pi diet using antibodies against NaPi-IIa **(A)**, full length Klotho **(B)**, Cyp24a1 **(C)** and Vdr **(D)**. The proteins of interest are shown in the top panels, whereas the corresponding β-actin signal is shown in the bottom image. Densitometric quantifications were normalized to the corresponding β-actin values. Bar graphs represent mean ± SD (n = 6/group) of the normalized values. Differences between both dietary groups were analyzed by unpaired student’s t-test with significant p values indicated as: ** p ≤ 0.01 and *** p < 0.0001.

Mice fed HPD had reduced protein abundance of the renal sodium-dependent phosphate cotransporter NaPi-IIa (Fig 3A). PTH as well as FGF23 (both increased in the HPD group) lower serum Pi by decreasing the amount of NaPi-IIa in brush border membrane of renal proximal tubule cells, thus suppressing renal Pi reabsorption [41–44]. Therefore, the low NaPi-IIa protein expression reflects the expected adaptations to the high Pi load. High FGF23 also decreases the renal expression of its co-receptor Klotho [45]. Accordingly mice fed HPD had lower renal Klotho protein expression compared to the LPD mice (Fig 3B). In agreement with the mRNA data, the protein expression of Cyp24a1 and Vdr in the kidney did not differ between the groups fed HPD or LPD (Fig 3C and 3D).

### FGF23 treatment does not affect the expression of vitamin D-related hydroxylases

**Fig 4:**
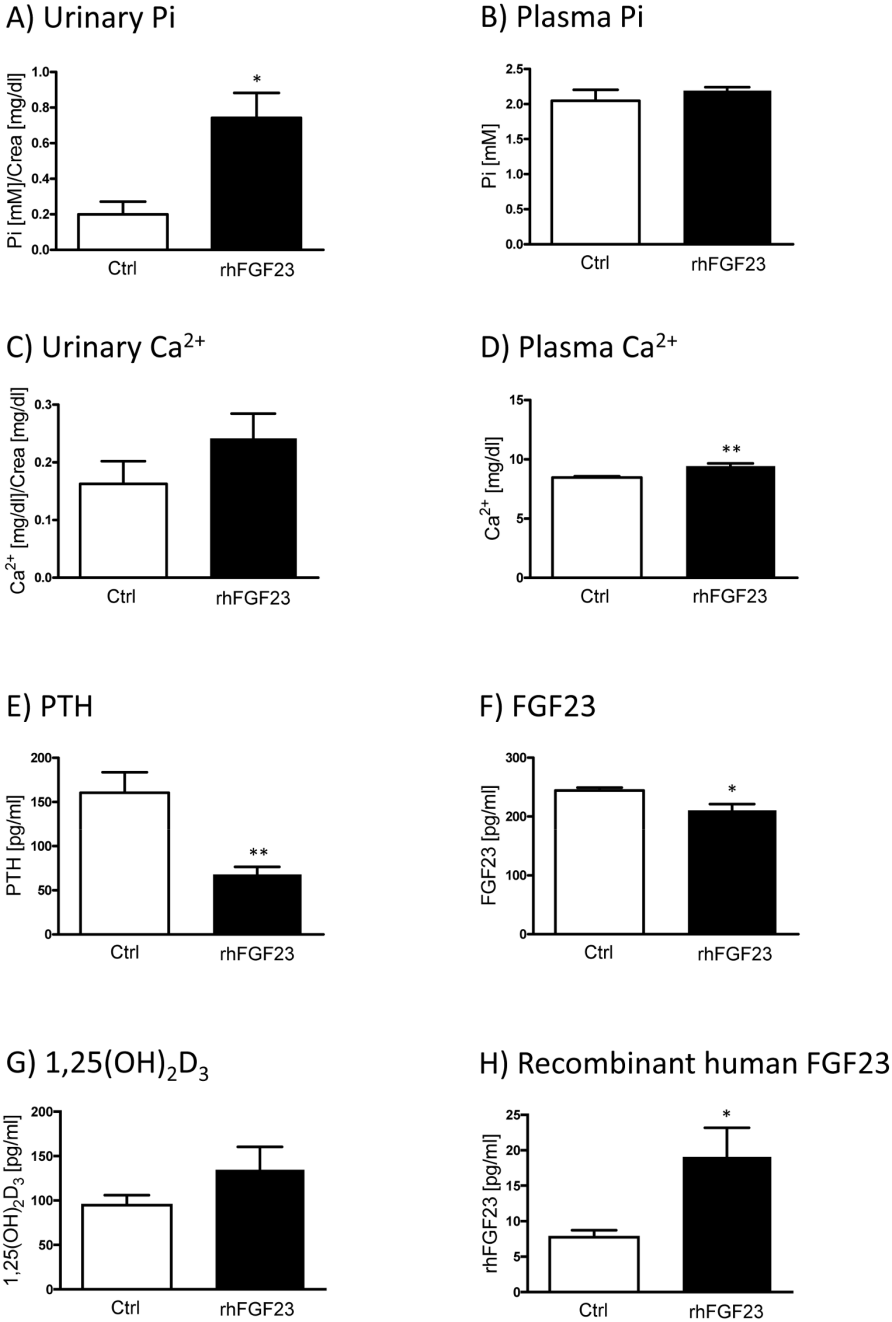
Effect of FGF23 administration on mineral and hormonal levels. Urinary and plasma concentrations of Pi **(A and B)** and Ca^2+^ **(C and D)** as well as plasma levels of PTH **(E)**, endogenous FGF23 **(F)**, 1,25(OH)_2_D **(G)** and recombinant human FGF23 **(H)** were measured in samples from mice injected with recombinant human FGF23 (rhFGF23) once a day for two consecutive days (black bars) and the respective vehicle treated controls (white bars). Graphs show mean ± SD (n = 5-6/group) and data was analyzed by unpaired student’s t-test with significant p values indicated as: * p ≤ 0.05 and ** p ≤ 0.01.

The injected recombinant human FGF23 (rhFGF23) triggered the expected phosphaturic effect, as mice injected with the hormone (once a day, for two consecutive days) exhibited a threefold higher urinary Pi excretion (Fig 4A). Plasma Pi levels were unaffected (Fig 4B). In this regard, it has been reported that single injections of FGF23 reduce plasma Pi [43]; however this effect was shown to be transient and in agreement with our data normophosphatemia was restored 24 hours after administration of FGF23. No significant changes in urinary Ca^2+^ excretion were observed (Fig 4C), whereas injections with rhFGF23 led to an increase in plasma Ca^2+^ levels compared to the control group (Fig 4D). In addition to its effect on Pi metabolism, FGF23 also influences renal Ca^2+^ reabsorption by increasing the expression of Ca^2+^ channels thereby enhancing Ca^2+^ transport in distal renal tubules [46]. These effects might explain the elevated serum Ca^2+^ levels but not the unchanged urinary excretion of Ca^2+^ found in our animals. As expected, mice treated with rhFGF23 had lower serum PTH concentrations (Fig 4E), as FGF23 downregulates the expression and secretion of PTH by the parathyroid [47]. Furthermore, injecting mice with rhFGF23 led to a significant decrease in plasma levels of endogenous intact FGF23 (Fig 4F), suggesting that the exogenous hormone triggered a negative feedback loop on the production of FGF23 by the mouse bone cells. rhFGF23 treatment did not affect serum 1,25(OH)_2_D_3_ levels significantly (Fig 4G). Although strong reductions on 1,25(OH)_2_D_3_ concentration were found in mice 9 hours post FGF23 administration, this effect was also transient with values normalized after 24 hours [43]. rhFGF23 was detected in mice injected with the recombinant protein even 24 hours after application (Fig 4H); its “detection” in control animals probably reflects antibody’s cross-reaction with endogenous mouse FGF23 (Fig 4H).

**Fig 5:**
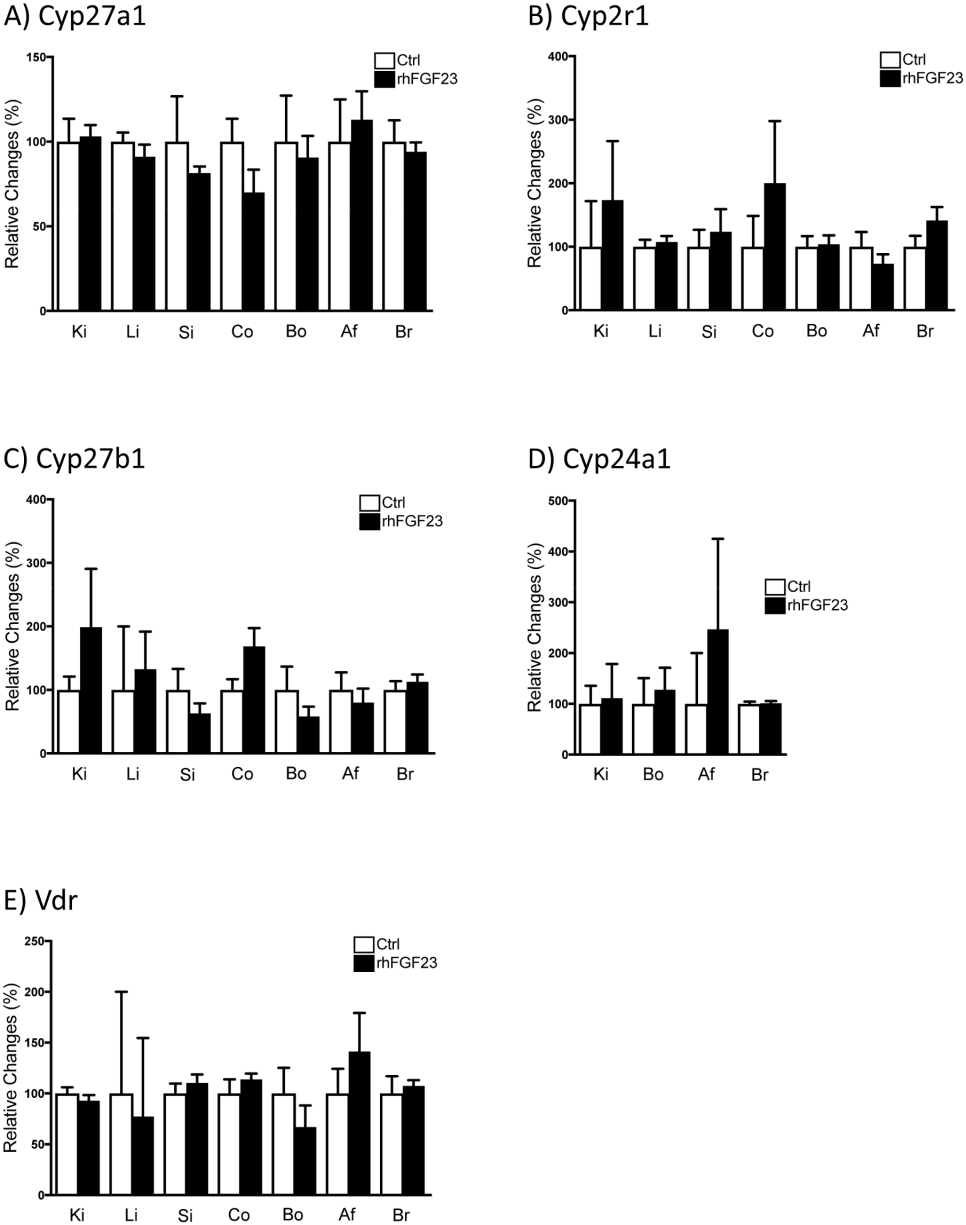
FGF23 did not alter the RNA expression of vitamin D-related hydroxylases and the Vdr. Gene expression of *Cyp27a1* **(A)**, *Cyp2r1* **(B)**, *Cyp27b1* **(C)**, *Cyp24a1* **(D)** and *Vdr* **(E)** was measured in kidney (Ki), liver (Li), small intestine (Si), colon (Co), bone (Bo), abdominal fat (Af) and brain (Br) samples of mice injected with recombinant human FGF23 (rhFGF23) once a day for two consecutive days (black bars) and the respective vehicle treated controls (white bars). Expression of the genes of interest was normalized to *Gapdh* (*Hprt* was used for normalization in bone). Graphs represent mean ± SD (n = 5-6/group) of relative changes, and data was analyzed by unpaired student’s t-test.

Surprisingly, rhFGF23 did not affect the mRNA expression of *Cyp27a1*, *Cyp2r1*, *Cyp27b1*, *Cyp24a1* nor *Vdr* in none of the analyzed organs (Fig 5A–5E). In this regard, lower Cyp27b1 and higher Cyp24a1 were reported in kidneys of mice shortly (1–9 hours) after FGF23 treatment, with values normalizing thereafter [43].

**Fig 6:**
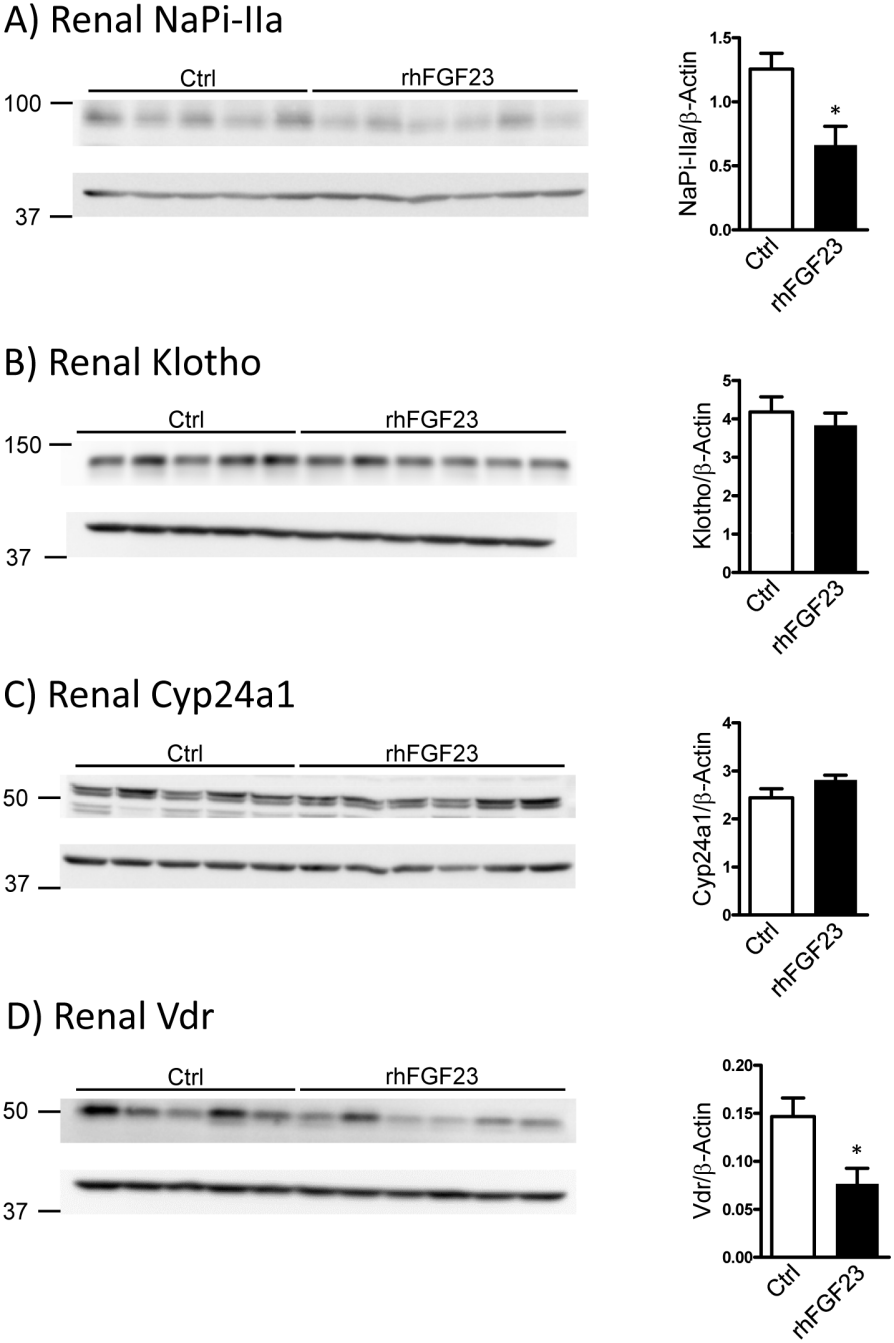
FGF23 treatment led to changes in renal NaPi-IIa and Vdr protein expression. Representative western blots of total renal protein homogenates from mice injected with either vehicle (Ctrl) or recombinant human FGF23 (rhFGF23) once a day for two consecutive days using antibodies against NaPi-IIa **(A)**, full length Klotho **(B)**, Cyp24a1 **(C)** and Vdr **(D)**. The proteins of interest are shown in the top panels, whereas the corresponding β-actin signal is shown in the bottom image. Densitometric quantification was normalized to the corresponding β-actin values. Bar graphs represent mean ± SD (n = 5-6/group) of the normalized values and data was analyzed by unpaired student’s t-test with significant p values indicated as: * p ≤ 0.05.

Mice injected with rhFGF23 had reduced renal NaPi-IIa protein expression (Fig 6A). This observation, together with hyperphosphaturia and reduced serum PTH levels indicates that the treatment induced the expected consequences on Pi homeostasis. However, and against expectations [45], the protein expression of Klotho (Fig 6B) was not affected by the rhFGF23 treatment. This absence of regulation may be eventually explained by the reduced levels of endogenous FGF23, probably counterbalancing the presence of rhFGF23, or by the additional regulation of klotho by other factors not controlled in this experiment. In agreement with the RNA data, the protein abundance of Cyp24a1 was similar in rhFGF23 and vehicle-injected animals (Fig 6C). Further experiments should be performed, testing whether different dosages or treatment lengths might lead to the expected effects in the renal hydroxylases. Interestingly, mice injected with rhFGF23 have lower Vdr protein expression in the kidney, compared to control animals (Fig 6D), suggesting that FGF23 may regulate the expression of Vdr in a vitamin D_3_ -independent way. Alternatively, a putative low 1,25(OH)_2_D_3_ at early time points may affect hydroxylases and Vdr with different time-courses.

### 1,25(OH)_2_D_3_ treatment regulates mainly the expression of its hydroxylases in the kidney

**Fig 7:**
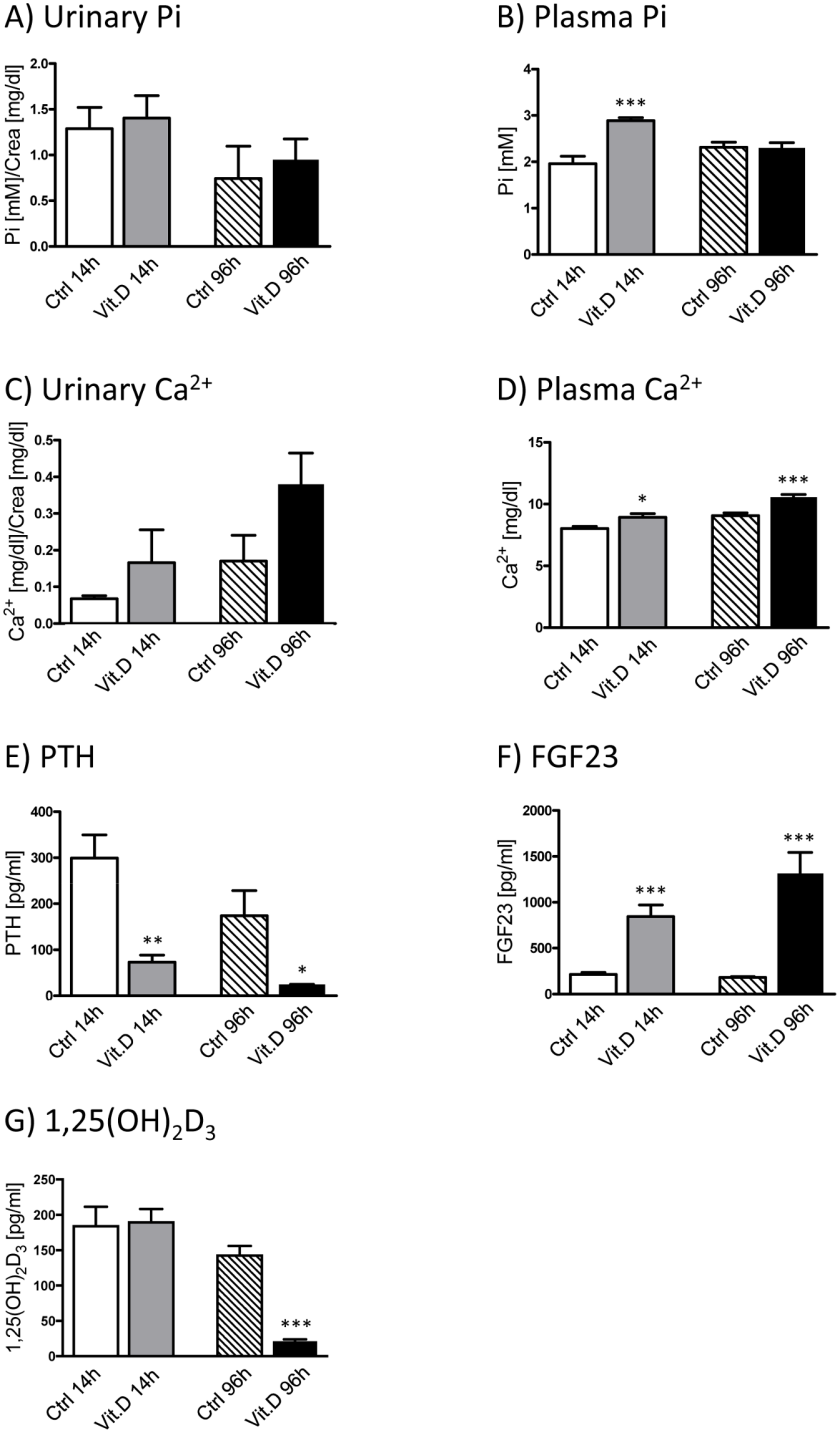
Effect of 1,25(OH)_2_D_3_ administration on mineral and hormonal levels. Urinary and plasma concentrations of Pi **(A and B)** and Ca^2+^ **(C and D)** as well as plasma levels of PTH **(E)**, FGF23 **(F)** and 1,25(OH)_2_D_3_ **(G)** were measured in samples from mice injected with 1,25(OH)_2_D_3_ either once within 14 hours (14h) or twice within 96 hours (96h) and their respective controls. Graphs show mean ± SD (n = 6/group) and data was analyzed by unpaired student’s t-test with significant p values indicated as: * p ≤ 0.05, ** p ≤ 0.01 and *** p < 0.0001.

1,25(OH)_2_D_3_ itself is an effective regulator of its own metabolism, suppressing CYP27B1 in kidney and inducing CYP24A1 in vitamin D_3_ target tissues [48, 49]. Therefore, the systemic response to 1,25(OH)_2_D_3_ administration was analyzed in short-term and long-term experiments. For both protocols, urinary Pi excretion did not differ between treated and the corresponding control groups (Fig 7A), while plasma Pi levels were increased in the short-term but no in long term experiments (Fig 7B). The latter results were expected, since vitamin D enhances intestinal Pi absorption, thereby resulting in a transient increase in plasma Pi, while also FGF23 is increased by 1,25(OH)_2_D_3_ counteracting an elevation of plasma Pi. 1,25(OH)_2_D_3_ further regulates the transcellular absorption of Ca^2+^ in epithelial cells of the intestine [50]. Although only a trend for higher urinary excretion was observed for the long-term treatment (Fig 7C), serum Ca^2+^ levels were elevated in both groups injected with 1,25(OH)_2_D_3_ (Fig 7D). 1,25(OH)_2_D_3_ inhibits the synthesis of PTH [24] whereas it increases the production of FGF23 [26], with the stimulated FGF23 potentially suppressing renal Pi reabsorption. Indeed, PTH levels were reduced whereas FGF23 concentration was increased upon short-term and long-term vitamin D administration (Fig 7E). Collectively, the above data suggest that 1,25(OH)_2_D_3_ administration induced the expected systemic responses, except for the absence of a hyperphosphaturic response especially in the long term protocol, where in addition to high FGF23 animals had normalized their plasma Pi values. Mice subjected to the long-term treatment had significantly lower serum 1,25(OH)_2_D_3_ levels compared to the control mice (Fig 7G), suggesting that after a certain treatment length the exogenous administration led either to a decrease of the endogenous production or to an increase in degradation; alternatively, the lower plasma levels may reflect increased binding of the hormone to the Vdr and subsequent internalization into target cells. Similar changes in plasma 1,25(OH)_2_D_3_ levels have been reported in 1,25(OH)_2_D_3_ treated mice [51].

**Fig 8:**
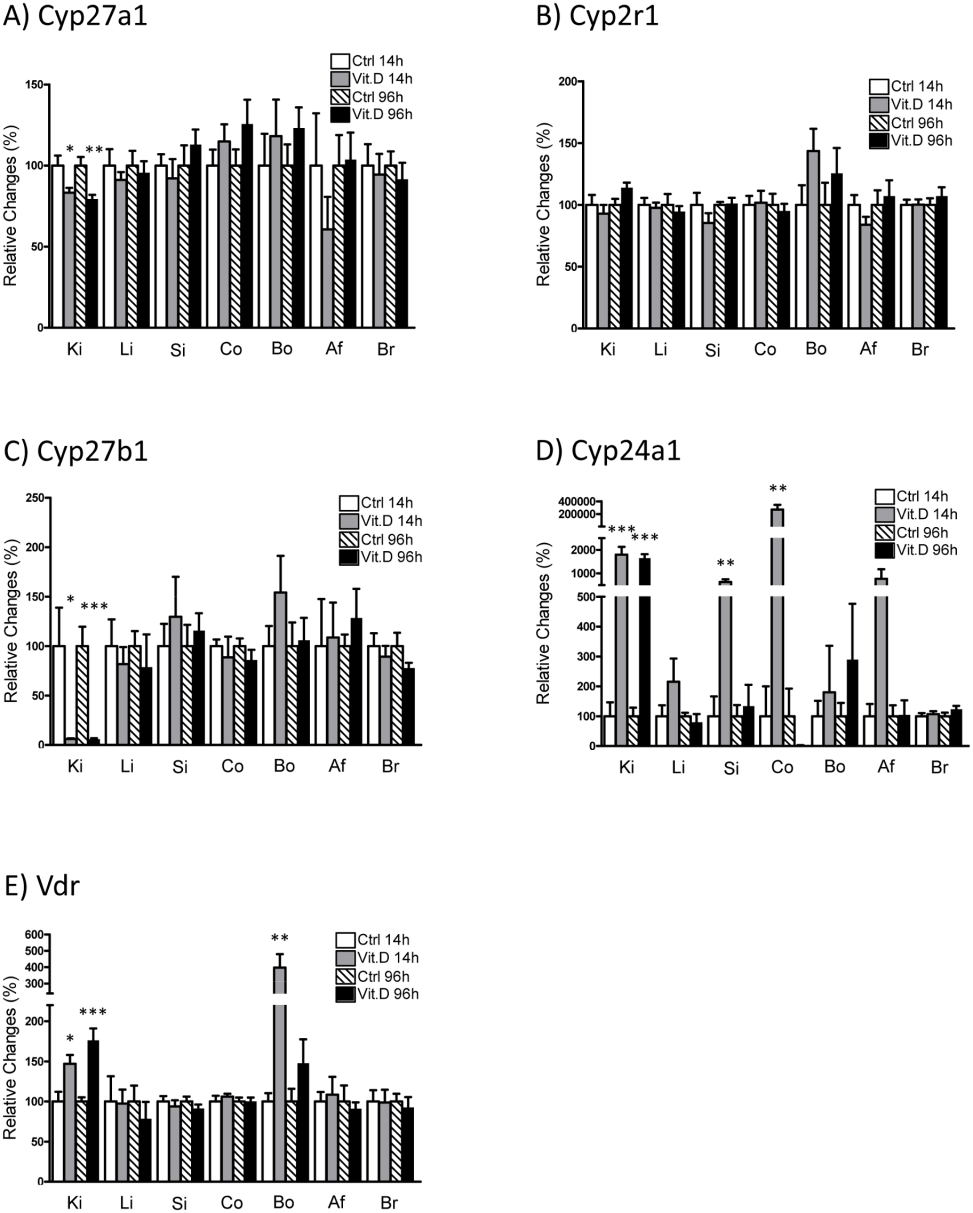
1,25(OH)_2_D_3_ treatment mainly changed renal mRNA expression. mRNA expression of *Cyp27a1* **(A)**, *Cyp2r1* **(B)**, *Cyp27b1* **(C)**, *Cyp24a1* **(D)** and *Vdr* **(E)** were measured in kidney (Ki), liver (Li), small intestine (Si), colon (Co), bone (Bo), abdominal fat (Af) and brain (Br) samples of mice injected with 1,25(OH)_2_D_3_ either once within 14 hours (14h, grey bars) or twice within 96 hours (96h, black bars) and their corresponding controls (white and striped bars, respectively). Expression of the mRNAs of interest was normalized to *Gapdh* (*Hprt* was used for normalization in bone). Graphs represent mean ± SD (n = 6/group) of relative changes (given as percentage and normalized to controls at 14 and 96 hrs, respectively) and data was analyzed by unpaired student’s t-test with significant p values indicated as: * p ≤ 0.05, ** p ≤ 0.01 and *** p < 0.0001.

The expression of *Cyp27a1* mRNA (detected in all analyzed organs) was exclusively regulated in kidney where both short and long-term treatment with 1,25(OH)_2_D_3_ led to a downregulation of the hydroxylase (Fig 8A). These changes suggest a potential feedback mechanism that suppresses the renal production of 25(OH)D_3_ in response to high 1,25(OH)_2_D_3_. Nevertheless, this assumption requires further investigations, since the detailed mechanisms of the regulation of *Cyp27a1* are unknown. Surprisingly, 1,25(OH)_2_D_3_ did not alter the hepatic expression of *Cyp27a1* (Fig 8A). In all tested organs, *Cyp2r1* expression remained unaffected by the two different 1,25(OH)_2_D_3_ applications (Fig 8B). In response to 1,25(OH)_2_D_3_ administration renal *Cyp27b1* was reduced whereas renal *Cyp24a1* expression was increased in both the short and long-term protocols (Fig 8C and 8D). In the long-term application these changes are consistent with the lower 1,25(OH)_2_D_3_ levels in plasma. Interestingly, both 1,25(OH)_2_D_3_ treatments failed to affect extrarenal *Cyp27b1* expression. As for the dietary and FGF23 studies, the expression of *Cyp24a1* was mainly detected in kidney under basal conditions and only at very low levels in the other organs analyzed. Treatment with 1,25(OH)_2_D_3_ increased *Cyp24a1* in the kidney as well as in small intestine and transiently in colon (Fig 8D). As for the dietary and FGF23 treatments, *Vdr* expression was detected in all tissues except in liver (S2 Table). Although the expression of the vitamin D receptor is expected to be upregulated by 1,25(OH)_2_D_3_ [40], the effects seem to be tissue and time dependent. Thus, renal mRNA expression of *Vdr* was increased for both protocols, whereas only transient changes were detected in bone (Fig 8E).

**Fig 9:**
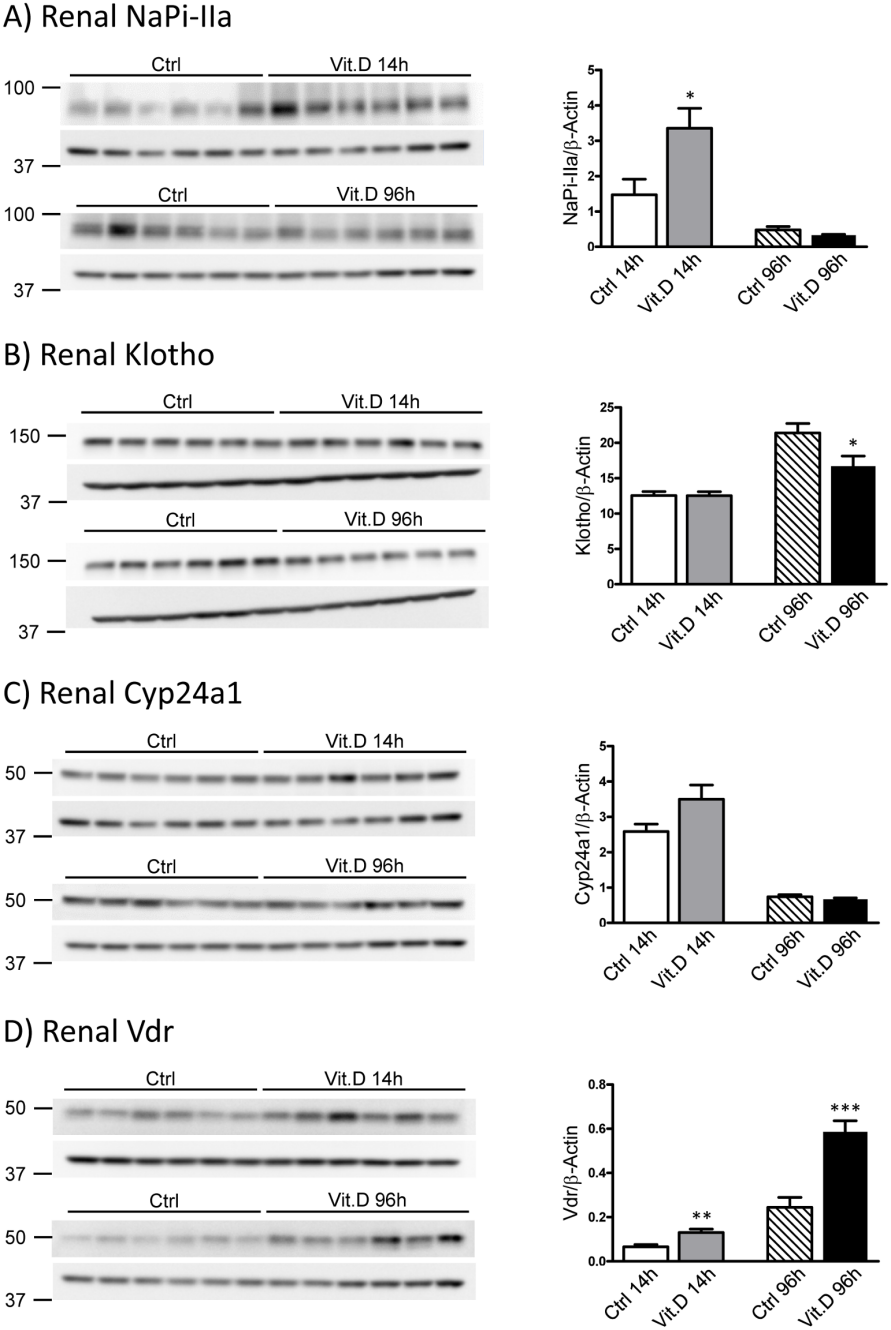
1,25(OH)_2_D_3_ administration regulated the protein abundance of NaPi-IIa, klotho and Vdr. Representative western blots of total renal protein homogenates from mice injected with 1,25(OH)_2_D_3_ either once within 14 hours (14h) or twice within 96 hours (96h) and the corresponding controls using antibodies against NaPi-IIa **(A)**, full length Klotho **(B)**, Cyp24a1 **(C)** and Vdr **(D)**. The proteins of interest are shown in the top panels, whereas the corresponding β-actin signal is shown in the bottom image. Densitometric quantification was normalized to the corresponding β-actin values. Bar graphs represent mean ± SD (n = 6/group) of the normalized values and data was analyzed by unpaired student’s t-test with significant p values indicated as: * p ≤ 0.05, ** p ≤ 0.01 and *** p < 0.0001.

Surprisingly, hyperphosphatemia and/or high FGF23 associated with 1,25(OH)_2_D_3_ administration did not correlate with reduced NaPi-IIa expression in total renal homogenates (Fig 9A). Thus, further experiments testing brush border membrane preparation should be performed to rule out that this apparent lack of regulation is due to membrane retrieval without complete NaPi-IIa degradation. 1,25(OH)_2_D_3_ has been shown to upregulate klotho mRNA in mouse kidney cell lines [52], however, in our *in vivo* experiments short term 1,25(OH)_2_D_3_ administration did not induce any changes in the protein expression whereas a reduction in renal klotho protein was observed in the long-term experiment (Fig 9B). This reduction was most probably provoked by the high FGF23 levels. The strong stimulation of the catabolizing enzyme Cyp24a1 on mRNA level by 1,25(OH)_2_D_3_ treatment did not result in higher protein abundance (Fig 9C). Further analysis of renal Cyp27b1 protein expression is clearly needed but hampered by the lack of specific antibodies. As it was already seen on mRNA level, renal protein expression of Vdr was increased upon short- or long-term treatment with 1,25(OH)_2_D_3_ (Fig 9D).

In summary, we have shown that: a) all analyzed genes involved in vitamin D metabolism show a wide tissue distribution, with the exception of the Cyp24a1, b) the regulation of their gene expression is tissue and treatment specific, c) chronic changes in dietary Pi mostly regulate the expression of 25-hydroxylases in liver, colon and abdominal fat, but these changes are not paralleled by altered levels of 1,25(OH)_2_D_3_ in plasma, d) short administration of FGF23 triggering major hormonal and mineral changes has no significant effects on vitamin D-regulating enzymes, suggesting different time courses for both actions, and e) 1,25(OH)_2_D_3_ treatment leads to major changes in renal hydroxylases.

## Supporting information

S1 Table Sequences of primers and probes used to quantify the mRNA expression of Cyp2r1, Cyp27a1, Cyp27b1 and Cyp24a1, Vdr, Gapdh and Hprt. (PDF)

S2 Table Relative mRNA expression of the Vdr, Cyp27a1, Cyp2r1, Cyp27b1 and Cyp24a1 in several tissues of mice fed high (HPD) and low phosphate diets (LPD) as well as of mice treated with FGF23 and vitamin D and their respective controls. Data was analyzed by unpaired student’s t-test with significant p values indicated as: * p ≤ 0.05, ** p ≤ 0.01 and *** p < 0.0001.
(PDF)
