# Supplementary material for: Regulation of vitamin D metabolizing enzymes in murine renal and extrarenal tissues by dietary phosphate, FGF23, and 1,25(OH)2D3
Source: PLoS One. 2018 May 17;13(5):e0195427. doi: 10.1371/journal.pone.0195427 (PMC5957386; doi:10.1371/journal.pone.0195427)
Supplement: S1 Table — (PDF) [file pone.0195427.s001.pdf]

S1 table

Gene	NCBI Ref. Sequence	Forward Primer<br>Reverse Primer	Probe<br>Reporter/Quencher
**Cyp2r1**	NM_177382.4	5'-AGC-AGA-GCC-GGG-TGT-ATG-3'<br>5'-CAC-TTT-GAT-GAA-CAA-GGC-ATT-C-3'	5'-TCT-TGG-AGG-CAT-ATC-AAC-TGT-CGT-TCT-3'<br>FAM/BHQ1
**Cyp27a1**	NM_024264	5'-GGA-CCG-GAA-CGC-TAC-AAT-TT3'<br>5'-CCA-AAG-GAG-GTT-GTC-CAC-AT-3'	5'-GCT-GCA-CTT-GCC-CGA-CCT-CC-3'<br>FAM/BHQ1
**Cyp27b1**	NM_010009.2	Mm01165916_g1, Thermo Fisher Scientific CH, FAM/NONE	
**Cyp24a1**	NM_009996.3	5'-CCA GCG GCT AGA GAT CAA AC-3'<br>5'-CAC GGG CTT CAT GAG TTT CT-3'	5'-TAC GGG CTG ATG ATC CTG GAA GGA C-3'<br>FAM/TAMRA
**Vdr**	NM_009504.4	5'-AGG CCC ACA CTC AGC TTC T-3'<br>5'-ACA GGT CCA GGG TCA CAG AG-3'	5'-TAC ACC CCC TCA CTG GAC ATG ATG G-3'<br>FAM/TAMRA
**Gapdh**	NM_008084.3	5'-GTC GTG GAT CTG ACG TGC C-3'<br>5'-GAT GCC TGC TTC ACC ACC TT -3'	5'-CCT GGA GAA ACC TGC CAA GTA TGA TGA CAT-3'<br>FAM/TAMRA
**Hprt**	NM_013556.2	5'-TTA TCA GAC TGA AGA GCT ACT GTA ATG ATC -3'<br>5'-TTA CCA GTG TCA ATT ATA TCT TCA ACA ATC -3'	5'-TGA GAG ATC ATC TCC ACC AAT AAC TTT TAT GTC CC-3'<br>VIC/NONE
