# Supplementary material for: Regulation of vitamin D metabolizing enzymes in murine renal and extrarenal tissues by dietary phosphate, FGF23, and 1,25(OH)2D3
Source: PLoS One. 2018 May 17;13(5):e0195427. doi: 10.1371/journal.pone.0195427 (PMC5957386; doi:10.1371/journal.pone.0195427)
Supplement: S2 Table — Data was analyzed by unpaired student’s t-test with significant p values indicated as: * p ≤ 0.05, ** p ≤ 0.01 and *** p < 0.0001. (PDF) [file pone.0195427.s002.pdf]

S2 table

		Vdr	Cyp27a1	Cyp2r1	Cyp27b1	Cyp24a1
**Kidney**	HPD	0.118748 ± 0.022677	0.007717 ± 0.001488	0.000487 ± 0.000477	0.010570 ± 0.007436	0.028468 ± 0.022766
	LPD	0.101655 ± 0.015833	0.006724 ± 0.000415	0.000205 ± 0.000202	0.021203 ± 0.020014	0.063462 ± 0.078547
	Ctrl	0.102975 ± 0.012625	0.007178 ± 0.001943	0.000146 ± 0.000209	0.004108 ± 0.001721	0.052249 ± 0.037171
	FGF23	0.095580 ± 0.012959	0.007408 ± 0.001062	0.000253 ± 0.000302	0.008152 ± 0.008464	0.058200 ± 0.078099
	Ctrl 14h	0.115856 ± 0.031392	0.030694 ± 0.004282	0.001826 ± 0.000328	0.019449 ± 0.016944	0.093049 ± 0.096715
	Vit.D 14h	*0.170294 ± 0.028782	*0.025546 ± 0.002086	0.001697 ± 0.000287	*0.001165 ± 0.000377	***1.671227 ± 0.702692
	Ctrl 48/96h	0.107605 ± 0.012864	0.025157 ± 0.003018	0.001938 ± 0.000210	0.009361 ± 0.004085	0.103019 ± 0.065919
	Vit.d 48/96h	***0.189814 ± 0.035203	**0.019990 ± 0.001438	0.002209 ± 0.000174	***0.000546 ± 0.000239	***1.695948 ± 0.388970
**Liver**	HPD	0.000040 ± 0.000042	0.222939 ± 0.051036	0.025714 ± 0.003499	0.000179 ± 0.000135	0.000000 ± 0.000000
	LPD	0.000018 ± 0.000026	*0.150058 ± 0.049398	***0.012636 ± 0.002322	0.000060 ± 0.000085	0.000000 ± 0.000000
	Ctrl	0.000013 ± 0.000025	0.183314 ± 0.019851	0.010695 ± 0.002324	0.000107 ± 0.000214	0.000000 ± 0.000000
	FGF23	0.000010 ± 0.000022	0.166971 ± 0.029378	0.011496 ± 0.002248	0.000142 ± 0.000142	0.000000 ± 0.000000
	Ctrl 14h	0.000044 ± 0.000031	0.672762 ± 0.151995	0.064149 ± 0.008049	0.000988 ± 0.000597	0.000013 ± 0.000010
	Vit.D 14h	0.000043 ± 0.000017	0.613031 ± 0.072818	0.062621 ± 0.005945	0.000809 ± 0.000380	0.000027 ± 0.000022
	Ctrl 48/96h	0.000049 ± 0.000022	0.618673 ± 0.125208	0.062262 ± 0.012303	0.000181 ± 0.000062	0.000017 ± 0.000004
	Vit.d 48/96h	0.000039 ± 0.000023	0.591673 ± 0.097459	0.058951 ± 0.006126	0.000143 ± 0.000134	0.000013 ± 0.000010
**Small intestine**	HPD	0.193209 ± 0.051530	0.006054 ± 0.003430	0.000570 ± 0.000304	0.000258 ± 0.000116	0.000105 ± 0.000134
	LPD	0.155145 ± 0.034091	0.007484 ± 0.002523	0.000409 ± 0.000243	*0.000106 ± 0.000053	0.000056 ± 0.000033
	Ctrl	0.389166 ± 0.075789	0.042353 ± 0.022754	0.000752 ± 0.000402	0.000480 ± 0.000318	0.000000 ± 0.000000
	FGF23	0.429946 ± 0.071130	0.034511 ± 0.003666	0.000929 ± 0.000598	0.000302 ± 0.000172	0.000000 ± 0.000000
	Ctrl 14h	0.324538 ± 0.048737	0.046249 ± 0.007280	0.004192 ± 0.000917	0.000250 ± 0.000126	0.000252 ± 0.000373
	Vit.D 14h	0.304412 ± 0.055790	0.042584 ± 0.012274	0.003579 ± 0.000739	0.000324 ± 0.000227	**0.001589 ± 0.000669
	Ctrl 48/96h	0.276544 ± 0.037542	0.019135 ± 0.005338	0.002536 ± 0.000136	0.000147 ± 0.000071	0.000012 ± 0.000010
	Vit.d 48/96h	0.252643 ± 0.030430	0.021636 ± 0.003933	0.002567 ± 0.000255	0.000169 ± 0.000058	0.000016 ± 0.000019
**Colon**	HPD	0.360090 ± 0.051826	0.117440 ± 0.037906	0.004038 ± 0.003510	0.002772 ± 0.001141	0.000000 ± 0.000000
	LPD	0.312218 ± 0.062334	*0.169197 ± 0.030358	0.002226 ± 0.001386	*0.001401 ± 0.000333	0.000000 ± 0.000000
	Ctrl	0.455389 ± 0.126159	0.022940 ± 0.006203	0.000855 ± 0.000828	0.000947 ± 0.000318	0.000000 ± 0.000000
	FGF23	0.517897 ± 0.059232	0.016056 ± 0.006872	0.001710 ± 0.001868	0.001595 ± 0.000612	0.000000 ± 0.000000
	Ctrl 14h	0.470758 ± 0.076336	0.028943 ± 0.006396	0.004589 ± 0.000746	0.001266 ± 0.000192	0.000002 ± 0.000004
	Vit.D 14h	0.499601 ± 0.037778	0.033251 ± 0.006821	0.004665 ± 0.001000	0.001121 ± 0.000594	**0.004897 ± 0.003030
	Ctrl 48/96h	0.362354 ± 0.039801	0.015036 ± 0.003347	0.004483 ± 0.000905	0.000968 ± 0.000168	0.000543 ± 0.001120
	Vit.d 48/96h	0.363353 ± 0.038089	0.018893 ± 0.005022	0.004263 ± 0.000577	0.000833 ± 0.000222	0.000014 ± 0.000007
**Bone**	HPD	0.687687 ± 0.224551	0.334211 ± 0.207254	0.032363 ± 0.015274	0.019866 ± 0.010252	0.000000 ± 0.000000
	LPD	0.509059 ± 0.168277	0.368574 ± 0.144314	0.023916 ± 0.011813	0.023247 ± 0.013567	0.000000 ± 0.000000
	Ctrl	0.470976 ± 0.237533	0.821665 ± 0.448098	0.015613 ± 0.005143	0.017982 ± 0.013241	0.000674 ± 0.000683
	FGF23	0.314885 ± 0.224593	0.744574 ± 0.235109	0.016208 ± 0.004873	0.010453 ± 0.006274	0.000859 ± 0.000660
	Ctrl 14h	0.248145 ± 0.058078	1.143491 ± 0.500968	0.019266 ± 0.006863	0.015628 ± 0.007126	0.000578 ± 0.000669
	Vit.D 14h	**0.987386 ± 0.459719	1.350262 ± 0.576690	0.027657 ± 0.007716	0.024096 ± 0.012985	0.001043 ± 0.002013
	Ctrl 48/96h	0.361022 ± 0.128742	1.100815 ± 0.320158	0.034888 ± 0.013960	0.023463 ± 0.012538	0.000807 ± 0.000808
	Vit.d 48/96h	0.533385 ± 0.240673	1.356283 ± 0.313469	0.043812 ± 0.015991	0.024871 ± 0.011808	0.002339 ± 0.003368
**Abdominal Fat**	HPD	0.003958 ± 0.005544	0.087243 ± 0.024206	0.007130 ± 0.002825	0.000559 ± 0.000264	0.000039 ± 0.000039
	LPD	0.000857 ± 0.000436	*0.057029 ± 0.012712	0.004194 ± 0.002383	0.000277 ± 0.000203	0.000067 ± 0.000106
	Ctrl	0.001645 ± 0.000801	0.112209 ± 0.055904	0.011291 ± 0.005211	0.000795 ± 0.000435	0.000004 ± 0.000008
	FGF23	0.002324 ± 0.001398	0.126752 ± 0.042129	0.008252 ± 0.003789	0.000637 ± 0.000394	0.000009 ± 0.000015
	Ctrl 14h	0.004761 ± 0.001277	0.042978 ± 0.030988	0.006979 ± 0.001231	0.009399 ± 0.010024	0.000014 ± 0.000013
	Vit.D 14h	0.005164 ± 0.002372	0.026060 ± 0.019302	0.005855 ± 0.001002	0.010220 ± 0.007419	0.000111 ± 0.000133
	Ctrl 48/96h	0.004322 ± 0.001931	0.042851 ± 0.018081	0.007785 ± 0.002044	0.000981 ± 0.000258	0.000018 ± 0.000015
	Vit.d 48/96h	0.003940 ± 0.000753	0.044473 ± 0.015839	0.008342 ± 0.002215	0.001260 ± 0.000649	0.000019 ± 0.000020
**Brain**	HPD	0.000296 ± 0.000066	0.006706 ± 0.001538	0.001259 ± 0.000557	0.001384 ± 0.000325	0.000219 ± 0.000020
	LPD	0.000377 ± 0.000068	0.008504 ± 0.001258	0.001044 ± 0.000341	0.001017 ± 0.000223	0.000224 ± 0.000053
	Ctrl	0.000289 ± 0.000097	0.007092 ± 0.001791	0.000776 ± 0.000267	0.001224 ± 0.000340	0.000189 ± 0.000017
	FGF23	0.000310 ± 0.000037	0.006667 ± 0.000861	0.001095 ± 0.000365	0.001379 ± 0.000324	0.000190 ± 0.000020
	Ctrl 14h	0.000230 ± 0.000072	0.005838 ± 0.001719	0.002614 ± 0.000248	0.001635 ± 0.000477	0.000072 ± 0.000016
	Vit.D 14h	0.000227 ± 0.000081	0.005515 ± 0.001663	0.002618 ± 0.000248	0.001462 ± 0.000397	0.000077 ± 0.000016
	Ctrl 48/96h	0.000242 ± 0.000052	0.006862 ± 0.001347	0.003218 ± 0.000385	0.001116 ± 0.000338	0.000078 ± 0.000021
	Vit.d 48/96h	0.000224 ± 0.000069	0.006283 ± 0.001565	0.003451 ± 0.000504	0.000868 ± 0.000135	0.000096 ± 0.000020
